# An Approach to the Uniform Dispersion of Graphene Nanosheets in Powder Metallurgy Nickel-Based Superalloy

**DOI:** 10.3390/ma12060974

**Published:** 2019-03-24

**Authors:** Yu-Xi Gao, Jin-Wen Zou, Xiao-Feng Wang, Jie Yang, Zhuo Li, Yan-Yan Zhu, Hua-Ming Wang

**Affiliations:** 1 School of Materials Science and Engineering, Beihang University, Beijing 100191, China; gaoyuxi@buaa.edu.cn (Y.-X.G.); lizhuo@buaa.edu.cn (Z.L.); wogayin@163.com (H.-M.W.); 2 National Engineering Laboratory of Additive Manufacturing for Large Metallic Components, School of Materials Science and Engineering, Beihang University, 37 Xueyuan Road, Beijing 100191, China; 3 AECC Beijing Institute of Aeronautical Materials, Beijing 100089, China; zoujw613@sina.com (J.-W.Z.); wangxiaofeng_0404@163.com (X.-F.W.); yangjient@163.com (J.Y.); 4 Science and Technology on Advanced High Temperature Structural Materials Laboratory, Beijing Institute of Aeronautical Materials, Beijing 100089, China

**Keywords:** powder metallurgical nickel-based superalloy, graphene nanosheets, PVA

## Abstract

In this paper, a wet-chemical based method was adopted to acquire the uniform dispersion of graphene nanosheets (GNSs) in a powder metallurgy nickel-based superalloy (FGH96) to fabricate a new GNSs reinforced FGH96 metal matrix composite. The surface of the FGH96 powder was modified using a hydrophilic surfactant named polyvinyl alcohol (PVA), which has good wettability and strong hydrogen bonding between the –OH groups of PVA and oxygen groups of GNSs such as –COOH, –CHO, and –OH. It was shown that the GNSs displayed much better dispersion uniformity on the PVA modified FGH96 powder than the unmodified one. The existence of PVA improved the adsorptive capacity of the GNSs attached on the powder surface and prevented the agglomeration in the following thermal preparation process. Consequently, the micro-hardness of PVA modified composite with 0.1 wt.% GNSs reached 497.9 HV, 3.4% higher than the unmodified FGH96 alloy. Therefore, this preparation process could act as the foundation of a common strategy for the fabrication of GNSs in metal matrix composites with good dispersion uniformity, which may have great potential application in aerospace applications.

## 1. Introduction

Graphene, a two-dimensional (2D) material with a film-like high aspect ratio [1,2,3], has a huge range of applications in chemistry, energy, bioscience and materials [4,5,6] given its unmatched structure and extraordinary mechanical performance such as excellent mechanical properties and outstanding processability [7]. In recent years, great efforts have been devoted to develop graphene nanosheet (GNS) reinforced metal matrix composites (MMCs). Most physical properties of metal matrix composites can be improved by a small amount of GNSs, for example, strength and toughness [8,9,10,11,12]. However, there are still some remaining unsolved problems for utilizing the maximum potential of GNSs such as the uniform dispersion of nanosheets and their interface with the matrix. Many methods including the ball milling process [13,14], laser assisted deposition [15,16], cold drawing [11], thermal spraying [17], and solvent dispersion [18] have been investigated to distribute GNSs uniformly over the metal matrix. Until now, the strength enhancement of the graphene-reinforced metal matrix composites has been lower than expected due to the agglomeration of GNSs in the metal matrix [8,19]. Therefore, it is valuable to develop an uncomplicated and effective preparation method to realize the full utilization of GNSs in the metal matrix.

Various fabrication techniques have been studied by previous researchers including wet chemical mixing, mechanical mixing, adsorption, and consolidation followed by hot isostatic pressing, sintering, rolling, or extrusion [20,21,22,23,24]. Although outstanding strength improvement in GNS reinforced metal matrix composites has been achieved, there are still some problems to be solved. For instance, the topography, structural integrity, and surface bonding properties of GNSs in the metal matrix are not satisfactory for the impact of the preparation process. Given the strong Van der Waals interaction between the GNSs and π–π interaction between the GNS layers, it is easy to form agglomeration and clusters, which can cause defects in a metal matrix. During the high temperature and pressure thermal process, GNSs may undergo a process of the complete destruction of original nanostructure, and thus cannot exert their excellent performance. In order to maximize the efficacy of GNSs, the agglomeration of the reinforcement phase should be minimized during the preparation process. Therefore, there are still tremendous challenges in the progress of a large-scale and economic application of GNS reinforced MMCs.

Among these fabrication techniques, the wet–chemical process followed by hot isostatic pressing (HIP) seems to be the best candidate [13,21,25] since the mechanical stirring forces can be used to break the interlayer bond between graphene nanosheets with less damage in a wet–chemical procedure, and the powder metallurgy method is suitable for the preparation of GNS reinforced MMCs. However, the powder surface is hydrophobic and incompatible with GNSs, and the agglomeration of GNSs usually results in a decrease in the bonding performance between GNSs and powder.

Polyvinyl alcohol (PVA) is a kind of non-toxic polymer that has been widely used as an adhesive, emulsifier, dispersant binder, and surfactant [26]. In order to solve the existing problems in the current popular preparation process, PVA has been used to introduce hydrophilic groups on the metal surface to improve surface compatibility with GNSs. In this study, the dispersion performance of GNSs on PVA-modified FGH96 powder was improved with the wet–chemical process followed by HIP. The PVA-modified powder exhibited attractive water wettability, combination, and compatibility with GNSs. Agglomeration of GNSs has been seldomly observed in the PVA-modified FGH96 powders. It was also found that GNSs and the metal matrix have a transition interface and excellent bonding properties. The testing result of microhardness indicated that the PVA modified FGH96 had a 3.4% improvement over the unmodified alloy. Due to the presence of PVA, this preparation process can achieve a homogeneous distribution and less agglomerations or destructions of GNSs in the powder.

## 2. Experimental

### 2.1. Raw Materials 

In the reinforcement phase, GNSs were first synthesized by Hummer’s method [27] from natural graphite, functionalized with carboxyl (–COOH), hydroxyl (–OH), aldehyde (–CHO), etc. The FGH96 powders were fabricated via gas atomization in a nitrogen atmosphere. PVA, with a molecular weight of about ~95,000 g/mol was used in the surface modification of the FGH96 nickel-based superalloy powders, supplied by Aladdin Agent Co. Ltd., Shanghai, China. The other reagents applied in this paper were of an analytical reagent grade.

### 2.2. Preparation of Composite Powders

**Figure 1:**
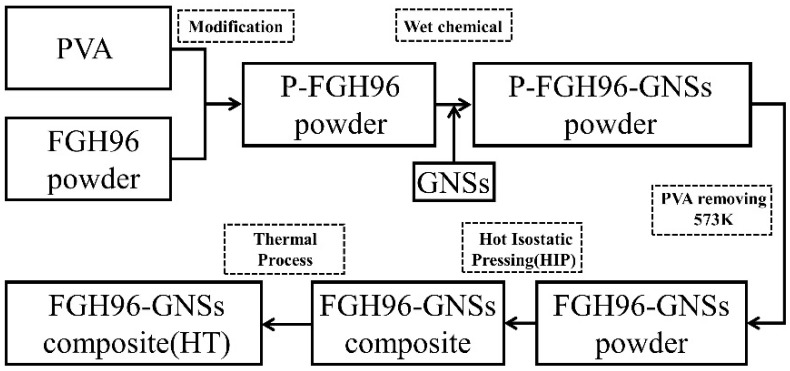
The flow diagram of the preparation process of the GNS reinforced PVA modified FGH96 composites.

Figure 1 illustrates the outline of the typical synthesis process of the PVA-modified FGH96 powder and composite. FGH96 powder was mixed with a 0.1 wt.% PVA aqueous solution (1000 mL highly purified deionized water), stirred at 60 °C for 1 h. Then, the as-prepared solution was filtered and washed to obtained the PVA-modified powder (P-FGH96). Next, 2 g of GNSs were thoroughly mixed with 1000 mL of deionized water to obtain a homogenous black dispersion with ultrasonicated stirring. P-FGH96 powder (2 kg) and the GNSs solution were added into deionized water together in a beaker. After that, the slurry mixture was mechanically stirred at 85 °C in an oil bath for 4 h until it reached a semi-dry state. The as-prepared composite powder was fully dried in a vacuum drying oven over 85 °C. In order to fully remove the residual PVA and the remaining graphene oxide thermally decomposed to GNSs, the P-FGH96 GNSs powder was followed with heat treatment in a vacuum environment at 500 °C for 14 h. Subsequently, the as-prepared mixtures were sealed in a stainless steel can, then pumped to 1 × 10^−3^ Pa. The steel can was further heated at 450 °C for 18 h and the moisture and remaining gas were removed from the mixed powder subsequently. The steel was hot isostatic pressed under 1150 °C and 150 MPa for 3 h. Finally, the composites were conducted with a 1150 °C solution heat treatment and 760 °C aging treatment.

### 2.3. Characterization

The microstructures and distributions of the GNSs within the FGH96 powder were characterized by field emission scanning electron microscopy (FE-SEM) (Hitachi S-4800, Hitachi, Ltd., Tokyo, Japan). High-resolution transmission electron microscopy (HR-TEM) and transmission electron microscopy (TEM) images were obtained with a 2100F field emission gun transmission electron microscope equipped with an energy-dispersive X-ray spectrometer (EDS) for elemental analyses. The TEM samples were fabricated by ion beam thinning. X-ray photoelectron spectroscopy (XPS) was conducted by an ESCAlab 250Xi electron spectrometer (Thermo Fisher Scientific, Waltham, MA, USA). Fourier transform infrared spectrometer (FT-IR) spectra were written down on a Bruker Vetex-70 IR spectrometer (Bruker, Karlsruhe, Germany). Raman spectra were measured with an ALMEGA-Dispersive Raman (ThermNicolet, Madison, WI, USA) under 532 nm laser excitation at room temperature. The average micro Vickers hardness of the composite, which was performed in the middle of the grain, was measured using a load of 0.2 kg for 10 s and the average of twenty test points was chosen. 

## 3. Results and Discussions

### 3.1. Morphology of GNSs and its Condition in Powder

**Figure 2:**
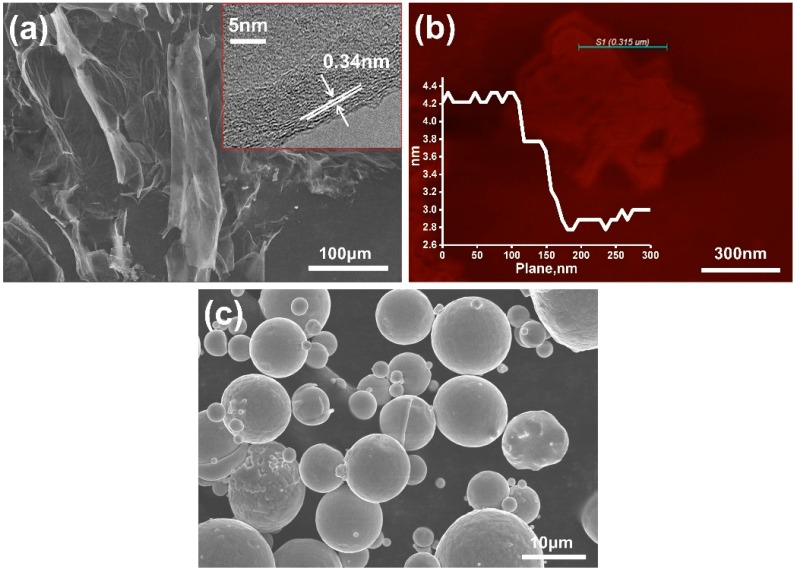
Microstructure of the GNSs and FGH96 powder used in the present paper. (**a**) SEM images of GNSs (the image in the upper right corner is the TEM image of GNSs with a 0.34 nm single layer thickness); (**b**) AFM images of the GNSs with a total thickness of 1.2–1.4 nm; (**c**) SEM images of the nickel-based superalloy with 53 μm.

Figure 2 present the microstructure of the GNSs and as-prepared gas-atomization FGH96 powder. It can be observed that the average thickness and diameters of GNSs were 1.2–1.4 nm and 50–100 μm, respectively. According to the single-layer-graphene thickness, the layers of the GNSs were about 10 to 15 with a large aspect ratio. The average diameter of the powder was about 53 μm and regularly spherical in shape with many dendrites on the surface.

**Figure 3:**
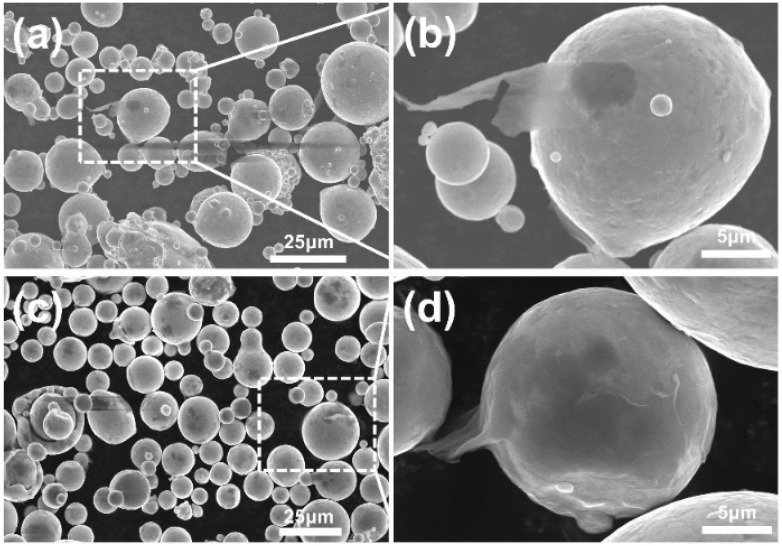
SEM images of the GNS reinforced FGH96 powder. (**a**,**b**) GNS reinforced unmodified FGH96 powders; (**c**,**d**) GNS reinforced PVA modified FGH96 powders.

**Figure 4:**
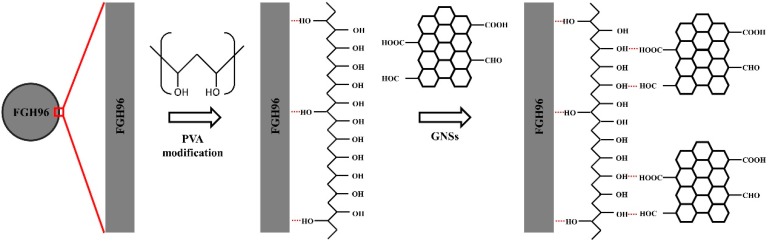
Diagram of the GNSs adsorption mechanism on the FGH96 powder surface.

Figure 3a,b shows the distributions of the GNSs with the unmodified FGH96 powders, revealing that only a few of the GNSs were connected onto the surface of the powders as most of the GNSs were agglomerated in the gaps between the FGH96 powders. As is known, the as-prepared GNSs functionalized with oxygen-rich groups are hydrophilic, while the FGH96 powder metal matrix is hydrophobic. The huge difference in wettability between the GNSs and FGH96 powder leads to a poor surface bonding performance between the two substances. Therefore, GNSs are more likely to agglomerate themselves into clusters rather than attach to the surface of the powder. In contrast, the PVA-modified FGH96 powders are illustrated in Figure 3c,d. As can be seen from the image, the GNSs were evenly distributed throughout the surface of the FGH96 powder. No significant agglomerations were observed between the powders, meaning that the FGH96 powder surface modified by PVA could greatly promote the uniform dispersion of GNSs. After modification by PVA, a thin hydrophilic film formed on the surface, which improved the surface wettability and made the GNSs easier to adhere to the surface. As illustrated in Figure 4, the amount of PVA on the surface of the FGH96 powder was largely increased due to the self-crosslinking adhesion of PVA after the surface modification. Due to the formation of hydrogen bonds between the –OH groups in PVA and the –COOH and –CHO groups of the GNSs, the PVA film on the powder surface was quite stable and difficult to detach from the powder surface via mechanical stirring and ultrasonication. The large quantity of –OH groups on the out-layer of the PVA films made the surface of the FGH96 powder change from hydrophobic to hydrophilic, with the PVA molecule chain extending into the solution. Then, the oxygen-rich group functionalized GNSs could be easily anchored on the surface of the FGH96 powder through hydrogen bonding. When using mechanical stirring, the remaining GNSs in the solution adhered to the surface of the FGH96 powder continuously. Furthermore, in the subsequent process, the attached GNSs were not easily dissociated, keeping a uniform dispersion statement. The PVA modification, which enhances water wettability and interactivity between the GNSs and metal matrix, is considered to be an effective way to promote dispersion uniformity.

### 3.2. Raman Spectroscopy

**Figure 5:**
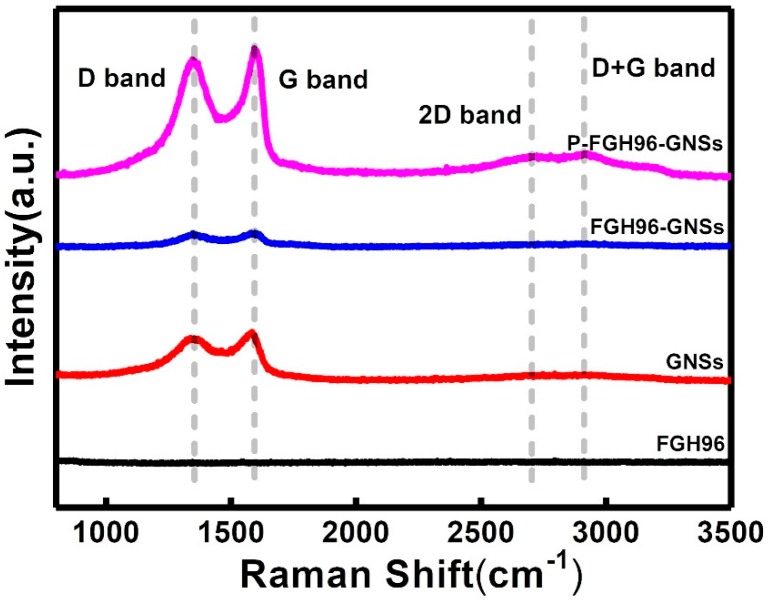
Raman spectra of the FGH96 powder, pristine GNSs, FGH96-GNSs powder, and P-FGH96-GNSs powder.

Figure 5 shows the Raman spectroscopy of GNSs, FGH96 powder, FGH96-GNSs powder, and P-FGH96-GNSs powder. The defect-induced peak (D-band, ~1350 cm^−1^), sp^2^-induced peak (G-band, ~1585 cm^−1^), and second order D peak (2D-band, ~2685 cm^−1^) are typical Raman spectrums of GNSs. In general, the relative intensities of the D- and G-bands can reflect the quality of a GNS sample. With the addition of PVA, a stronger absorption peak (~2697 cm^−1^) shift in the 2D-band was observed in the powder mixture than in the GNS data curve, which may be caused by the fact that the existence of PVA makes the GNSs have a more excellent stripping effect. Moreover, adding PVA makes the G band (~1581 cm^−1^) for pure GNSs turn to higher wave-numbers (~1591 cm^−1^), which is due to the strong π–π interactions between the GNSs and PVA. The intensity ratio (*I_D_/I_G_*), which is related to the defect in graphite peaks, is generally used to measure the amount of damage to the GNS structure. The higher intensity ratio (*I_D_/I_G_*) indicates an improvement in the number of defects and disorders in the GNSs. After calculation, it was found that ratio (*I_D_/I_G_*) values corresponding to the GNSs, FGH96-GNSs, and P-FGH96-GNSs samples were 0.908, 0.912, and 0.950, respectively. Additionally, a slight upward trend was observed, which suggested hydrogen-bonding crosslinking of PVA with the GNSs. The binding sites and vacancies that occurred when the GNSs were connected to the powder surface led to defects arising after the mixing procedure. However, due to the macromolecular chain interaction effect of PVA, the structural integrity of the GNSs remained unchanged during the wet–chemical mixing method.

### 3.3. Fourier Transform Infrared (FT-IR) Analysis

**Figure 6:**
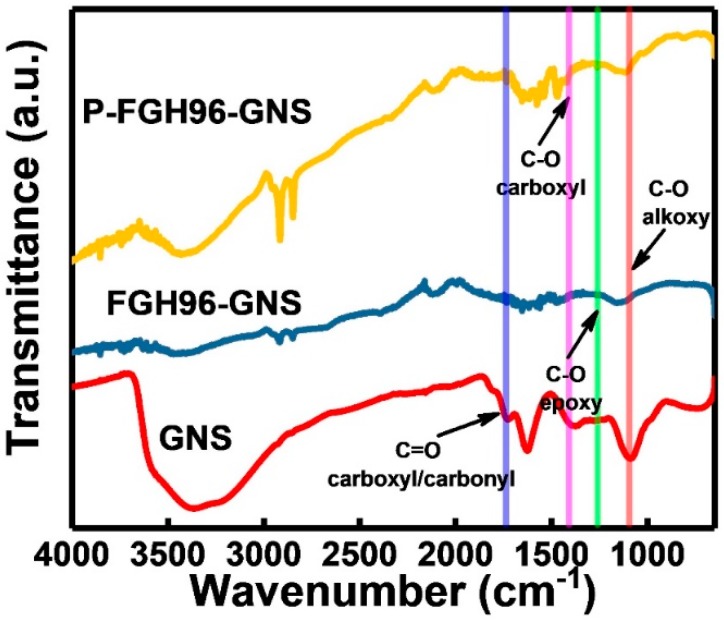
FTIR spectra of the pristine GNSs, FGH96-GNSs powder, and P-FGH96-GNSs powder.

Figure 6 shows the FTIR spectra of the GNSs, FGH96-GNSs powder, and P-FGH96-GNSs powder. The stretching vibration of –OH at 3450 cm^−1^, C–H at 2928 cm^−1^, –COO^–^ at 1589 cm^−1^, and 1419 cm^−1^ are indicated as the characteristic peaks of the GNSs. There exists the typical absorption peaks of oxygen-containing functional groups in the GNSs, a vibration peak at 1730 cm^−1^ represents the C=O group; a peak at 1420 cm^−1^ indicates the existence of the C–O group, and peaks at 1230 and 1050 cm^−1^ relate to the epoxide and alkoxy groups, respectively. It can be seen that with the introduction of PVA, the O–H bend vibration of the adsorbed water at 1640 cm^−1^; C–H stretch and bend vibrations at 2850, 2925, and 1460 cm^–1^, and O–H stretch and bend vibrations at 3425 and 1380 cm^−1^ showed stronger absorption peaks. Additionally, the band at 1100 cm^−1^ indicated the characteristic peak of PVA, which is related to the C–O stretch vibration. Compared with the powder without PVA modification, the characteristic peaks at 2865 cm^−1^ and 2935 cm^−1^ of the FTIR spectrum of the PVA modified GNSs/FGH96 powder represent the symmetry and asymmetry stretching vibration of the alkyl groups, affirming the way of the π–π cross-linking via the PVA-GNSs molecules was achieved. These results are further evidence of the successful introduction of PVA into the powder to obtain GNSs evenly distributed into the powder with strong interaction.

### 3.4. X-Ray Photoelectron Spectroscopy (XPS) Analysis

**Figure 7:**
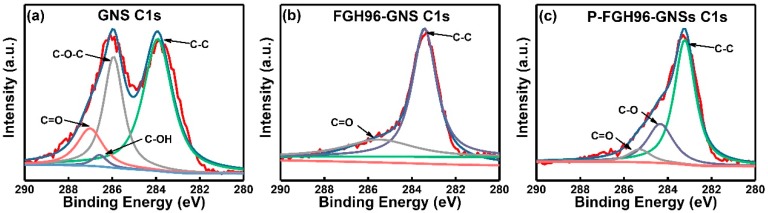
C 1s XPS spectrum of the pristine GNSs (**a**), FGH96-GNSs powder (**b**), and P-FGH96-GNSs powder (**c**).

Figure 7a–c indicate the XPS of the C 1s characteristic peaks of GNSs, FGH96-GNSs powder, and P-FGH96-GNSs powder, respectively. The XPS peaks of GNSs showed some types of carbon bonds: 289 eV for C(O)O, 288 eV for C=O, 286 eV for C–O (epoxy/ether), 285 eV for C–C, and 284 eV for C–OH. Compared with the peaks of GNSs in the FGH96 powder, the C 1s results of the PVA modified powder showed an increasing dosage of the epoxy/ether group, indicating the formation of intermolecular hydrogen bonds and excellent compatibility between the GNSs and the PVA film.

### 3.5. Microstructure

**Figure 8:**
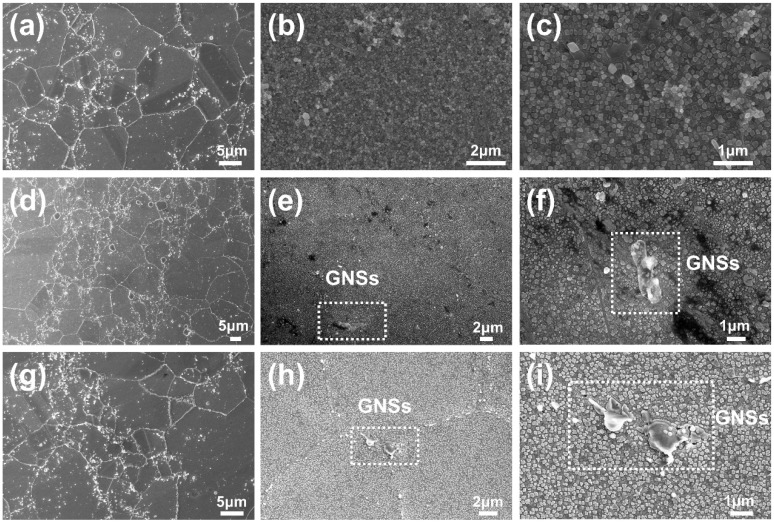
SEM observations for the FGH96 metal matrix: (**a**) Chemically etched and (**b**,**c**) electrochemically etched. GNSs reinforced FGH96 metal matrix composite: (**d**) Chemically etched and (**e**,**f**) Electrochemically etched. GNSs reinforced PVA modified FGH96 metal matrix composite: (**g**) for Chemically etched and (**h**,**i**) Electrochemically etched.

The SEM images of the FGH96, GNSs reinforced FGH96, and GNSs reinforced PVA modified FGH96 are shown in Figure 8a–i. As shown in Figure 8a,d,g, all samples had a similar grain size, which was about 25 μm. The addition of GNSs did not significantly affect the γ and γ’ phase, as shown in Figure 8b,e,h. Figure 8f shows that the GNSs dispersed around the FGH96 matrix. It is highly possible that the agglomeration of GNSs will occur with no PVA. In addition, the PVA modified FGH96 composite displayed a microstructure with a homogeneous dispersion of GNSs in the matrix compared with Figure 8f,i. With the modification of PVA, the GNSs exhibited a more flattened and less agglomerated structural characteristic in the metal matrix. The GNSs could be dispersed in the matrix with less agglomeration because the GNSs were evenly dispersed by the PVA modification and as there were many interactions between the powder surface and the PVA, the GNSs were more likely to adhere to the surface of the powder. Therefore, the GNSs could still retain their original morphology during the subsequent thermal process.

### 3.6. Transmission Electron Microscopy (TEM) Analysis

**Figure 9:**
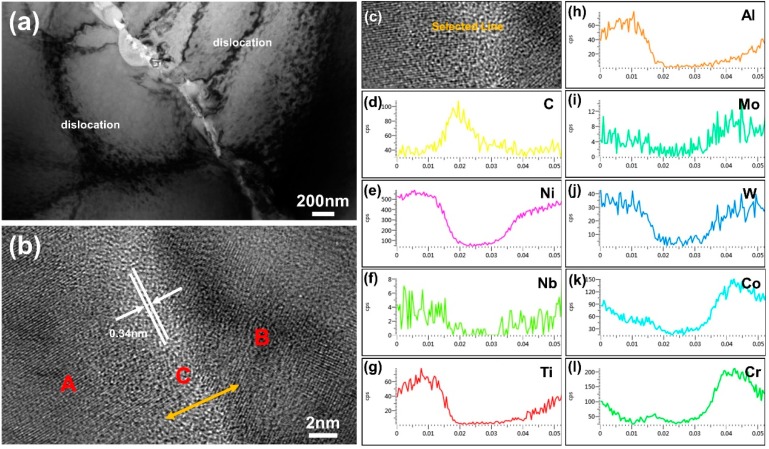
TEM image of PVA modified 0.1 wt.% GNS reinforced FGH96 composite along the boundary: (**a**) Bright-field image showing the interface of GNSs and nickel-based superalloy matrix; (**b**) HRTEM image of the boundary in Figure 7a; (**c**–**l**) EDS line scanning spectrum of the chemical element composition of the matrix composites.

Figure 9 depicts the TEM micrographs of the GNS distribution and PVA modified GNS reinforced FGH96 metal matrix interfacial states. As shown in Figure 9a, clustered GNSs were found in the PVA modified GNS reinforced FGH96 composites after the HIP and heat treatment process. GNSs were found to be uniformly dispersed along the grain boundary in the composite and no obvious agglomeration was observed. Furthermore, a high-density dislocation structure was found after the thermal process around the grain boundary. The GNSs maintained their original layer structure between the grain boundaries; the size of the lattice fringes of the GNSs monolithic layer in the composites was ~0.34 nm, which agreed with an interplanar spacing of (0002) of graphite as verified by the HRTEM images (Figure 9b). The total thickness of the graphene interlayer was approximately 1.1–1.2 nm, thinner than the data in Figure 2a,b, which may be due to the existence of PVA acting as spacers efficiently prevents GNSs from re-stacking and re-agglomeration, leading to the uniform distribution of GNSs in the FGH96 metal matrix. Furthermore, the surface modification agent PVA greatly improved the compatibility and interfacial bonding between the GNSs and FGH96 metal matrix. Excellent surface bonding properties are expected to improve the mechanical properties of PVA modified GNSs/FGH96. Moreover, the distribution of the main elements was carried out via line scan by the energy spectrum of TEM. The corresponding EDS line scanning image revealed the distribution of the main elements of FGH96 and carbon in the GNSs along the interface at the grain boundary transition area (as illustrated in Figure 9c–l). The metal matrix phase region (marked as A and B) was mainly composed of metal base elements such as Ni, Ti, Al, Cr, and so on. However, in the intermediate region (marked as C), it was mainly composed of carbon elements from the GNSs. From the metal matrix phase region to the middle area, it was found that the content of the C element gradually increased and presented a mountain-like curve, while the peak of the metal elements appeared as a canyon-shape. This indicates that there is a transition interface between the GNSs and metal matrix, which may be caused by the existence of interface reaction and diffusion.

### 3.7. Microhardness of the Composites

**Figure 10:**
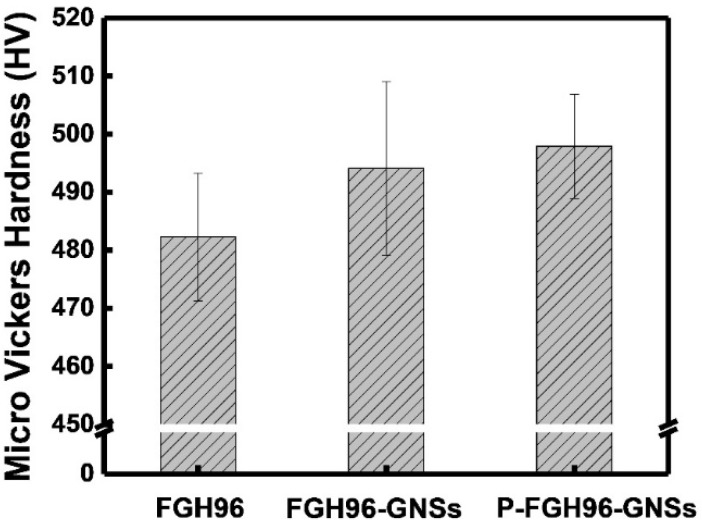
The microhardness of the matrix FGH96, 0.1 wt.% GNSs/FGH96, and PVA modified 0.1 wt.% GNSs/FGH96 composites, respectively.

The microhardness test revealed that the PVA modified process was beneficial in improving the mechanical properties of the fabricated composites. The microhardness of the FGH96 alloy, GNSs reinforced FGH96 alloy, and PVA modified GNSs reinforced FGH96 alloy are shown in Figure 10. The microhardness of the GNSs reinforced FGH96 composites with 0.1 wt.% GNSs was 494.1 HV, and the GNSs reinforced PVA modified FGH96 composites reached 497.9 HV, showing a 2.6% and 3.4% increase over unreinforced FGH96 under the same thermal process conditions. It is speculated that the improvement in hardness may be attributed to the existence of GNSs, which act as a hard reinforcement phase in the prepared MMCs. The microhardness of the PVA modified GNS reinforced FGH96 composites improved because the presence of PVA led to the uniform distribution of GNSs in the metal matrix. It was considered that the increase in microhardness may be due to the following reasons. First, the GNSs in the P-FGH96-GNSs powder presented a better dispersibility and compatibility with the FGH96 powder than the GNSs in the FGH96-GNSs powder (Figure 3). If there is agglomeration of the GNSs in the composite, pores will appear in the subsequent thermal process of the composite material, thus reducing the mechanical properties of the material. Second, it is highly possible that the interaction between the PVA and GNSs has a direct influence on improving the mechanical properties. The presence of PVA changes the wettability of the powder surface, and the interaction between PVA and GNSs is more obvious because of this long-chain macromolecular structure. The interactive effect came from the intermolecular hydrogen bonding crosslinking (as demonstrated by Raman spectroscopy) and ionic bonding crosslinking (as demonstrated by FT-IR and XPS spectroscopy), which led to a superior uniform dispersion of the GNSs on the surface of the powder, which favors improving the hardness of the prepared MMCs. Third, the interface between the GNSs and composite material was clean and tidy (as shown in Figure 9) and without the formation of defects and micropores. It is generally believed that the combination of a tight interface is conducive to limiting the movement of dislocations and acting as a carrier at the same time. Therefore, a combination of these reasons led to an increase in the microhardness of the PVA modified composite. A reinforcing effect is obtained with the GNSs uniformly dispersed in the metal matrix, which is beneficial to strengthening mechanisms such as the Orowan and grain refinement strengthening effect and synergy with the GNSs acts as a hard particle strengthening agent. Further experiments will concentrate on the effect of the GNSs content on the mechanical properties and GNSs strengthening mechanism 

The advantage of the wet–chemical approach is that by regulating the surface wettability of the powder with PVA is to make the GNSs easy to disperse uniformly on the powder surface. Additionally, in the PVA modified wet–chemical process, only water is added as a solvent. Therefore, it is an excellent method for fabricating GNS reinforced nickel-based superalloy composites because of its easy-preparation, high-efficiency, energy-saving, and environmental-protection advantages. Furthermore, this PVA surface modification method can be used on other metal matrix composites such as Ti, Cu, and Mg to improve the surface hydrophobic properties of the metal powder to achieve a better interaction with the GNSs.

## 4. Conclusions

In summary, this work revealed a novel method to improve the uniformity of GNSs in FGH96 powders. The great promotion in the uniform dispersion of GNSs on FGH96 largely relies on the modification of the hydrophilic PVA. The introduction of PVA can form a hydrophilic film on the surface of the powder, thus improving the water wettability and adhesion ability via powerful hydrogen bonding and the π–π interaction between the PVA and GNSs, which makes it possible to produce a highly uniform dispersion without the agglomeration of GNSs. Furthermore, the PVA modified process promotes the microhardness of the fabricated MMCs as the PVA modified GNSs-FGH96 composite showed a 3.4% increment over unreinforced FGH96 after the thermal process, which was decided by the high quality of the GNSs and its forceful interfacial bonding with the metal matrix. Therefore, this economical, feasible, and controllable method has great application potential for the fabrication of other GNS-distributed MMCs with enhanced properties, which can be expected to be applied in aerospace applications.

## References

[1] Geim A.K. (2009). Graphene: Status and Prospects. Science.

[2] Geim A.K., Novoselov K.S. (2007). The rise of graphene. Nat. Mater..

[3] Novoselov K.S., Geim A.K., Morozov S.V., Jiang D., Zhang Y., Dubonos S.V., Grigorieva I.V., Firsov A.A. (2004). Electric Field Effect in Atomically Thin Carbon Films. Science.

[4] Georgakilas V., Tiwari J.N., Kemp K.C., Perman J.A., Bourlinos A.B., Kim K.S., Zboril R. (2016). Noncovalent Functionalization of Graphene and Graphene Oxide for Energy Materials, Biosensing, Catalytic, and Biomedical Applications. Chem. Rev..

[5] Huang X., Qi X., Boey F., Zhang H. (2012). Graphene-based composites. Chem. Soc. Rev..

[6] Kong X., Liu Q., Zhang C., Peng Z., Chen Q. (2017). Elemental two-dimensional nanosheets beyond graphene. Chem. Soc. Rev..

[7] Lee C., Wei X., Kysar J.W., Hone J. (2008). Measurement of the Elastic Properties and Intrinsic Strength of Monolayer Graphene. Science.

[8] Wang M., Zhao Y., Wang L.-D., Zhu Y.-P., Wang X.-J., Sheng J., Yang Z.-Y., Shi H.-L., Shi Z.-D., Fei W.-D. (2018). Achieving high strength and ductility in graphene/magnesium composite via an in-situ reaction wetting process. Carbon.

[9] Zhou W., Yamaguchi T., Kikuchi K., Nomura N., Kawasaki A. (2017). Effectively enhanced load transfer by interfacial reactions in multi-walled carbon nanotube reinforced Al matrix composites. Acta Mater..

[10] Guo B., Zhang X., Cen X., Chen B., Wang X., Song M., Ni S., Yi J., Shen T., Du Y. (2018). Enhanced mechanical properties of aluminum based composites reinforced by chemically oxidized carbon nanotubes. Carbon.

[11] Li J., Zhang X., Geng L. (2018). Improving graphene distribution and mechanical properties of GNP/Al composites by cold drawing. Mater. Des..

[12] Morris B., Becton M., Wang X. (2018). Mechanical abnormality in graphene-based lamellar superstructures. Carbon.

[13] Yan S.J., Dai S.L., Zhang X.Y., Yang C., Hong Q.H., Chen J.Z., Lin Z.M. (2014). Investigating aluminum alloy reinforced by graphene nanoflakes. Mater. Sci. Eng. A.

[14] Xu R., Tan Z., Xiong D., Fan G., Guo Q., Zhang J., Su Y., Li Z., Zhang D. (2017). Balanced strength and ductility in CNT/Al composites achieved by flake powder metallurgy via shift-speed ball milling. Compos. Part A Appl. S.

[15] Yamanaka S., Gonda R., Kawasaki A., Sakamoto H., Mekuchi Y., Kuno M., Tsukada T. (2007). Fabrication and Thermal Properties of Carbon Nanotube/Nickel Composite by Spark Plasma Sintering Method. Mater. Trans..

[16] Hu Z., Chen F., Xu J., Nian Q., Lin D., Chen C., Zhu X., Chen Y., Zhang M. (2018). 3D printing graphene-aluminum nanocomposites. J. Alloy. Compd..

[17] Cao M., Xiong D.-B., Tan Z., Ji G., Amin-Ahmadi B., Guo Q., Fan G., Guo C., Li Z., Zhang D. (2017). Aligning graphene in bulk copper: Nacre-inspired nanolaminated architecture coupled with in-situ processing for enhanced mechanical properties and high electrical conductivity. Carbon.

[18] Sahoo B., Joseph J., Sharma A., Paul J. (2017). Surface modification of aluminium by graphene impregnation. Mater. Des..

[19] Zhou W., Fan Y., Feng X., Kikuchi K., Nomura N., Kawasaki A. (2018). Creation of individual few-layer graphene incorporated in an aluminum matrix. Compos. Part A Appl. S.

[20] Bisht A., Srivastava M., Kumar R.M., Lahiri I., Lahiri D. (2017). Strengthening mechanism in graphene nanoplatelets reinforced aluminum composite fabricated through spark plasma sintering. Mater. Sci. Eng. A.

[21] Cao Z., Wang X., Li J., Wu Y., Zhang H., Guo J., Wang S. (2017). Reinforcement with graphene nanoflakes in titanium matrix composites. J. Alloy. Compd..

[22] Jiang J., He X., Du J., Pang X., Yang H., Wei Z. (2018). In-situ fabrication of graphene-nickel matrix composites. Mater. Lett..

[23] Li Z., Guo Q., Li Z., Fan G., Xiong D.-B., Su Y., Zhang J., Zhang D. (2015). Enhanced Mechanical Properties of Graphene (Reduced Graphene Oxide)/Aluminum Composites with a Bioinspired Nanolaminated Structure. Nano Lett..

[24] Liu J., Khan U., Coleman J., Fernandez B., Rodriguez P., Naher S., Brabazon D. (2016). Graphene oxide and graphene nanosheet reinforced aluminium matrix composites: Powder synthesis and prepared composite characteristics. Mater. Des..

[25] Li J.L., Xiong Y.C., Wang X.D., Yan S.J., Yang C., He W.W., Chen J.Z., Wang S.Q., Zhang X.Y., Dai S.L. (2015). Microstructure and tensile properties of bulk nanostructured aluminum/graphene composites prepared via cryomilling. Mater. Sci. Eng. A.

[26] Wang J., Cheng Q., Lin L., Jiang L. (2014). Synergistic Toughening of Bioinspired Poly(vinyl alcohol)–Clay–Nanofibrillar Cellulose Artificial Nacre. ACS Nano.

[27] Hummers W.S., Offeman R.E. (1958). Preparation of Graphitic Oxide. J. Am. Chem. Soc..

